# Pathway-Level Synthesis Reveals Contrast-Dependent Rat Brain Transcriptomic Responses to Alcohol

**DOI:** 10.3390/ijms27198873

**Published:** 2026-10-05

**Authors:** Hongrun An, Jie Yu, Chunxue Shao, Deyu Meng, Guang Yang, Ziheng Wang

**Affiliations:** Division of Computational Biology, Chinese Center of Exercise Epidemiology, Northeast Normal University, Changchun 130024, China

**Keywords:** ethanol, transcriptomics, pathway analysis, withdrawal, brain region, sensitivity analysis, meta-analysis

## Abstract

Public rat brain transcriptomic studies differ in sampled region and alcohol paradigm, which complicates assessment of reproducible molecular responses. We assembled 12 datasets, including four primary region-resolved datasets (64 samples) from the hippocampus, ventral tegmental area, amygdala, and nucleus accumbens. Each dataset was analyzed separately and projected onto predefined addiction-related, exercise-related, and stress-related pathway axes; only aligned contrast-level effects were synthesized across datasets. In the closest available two-dataset comparison, both studies used adult male Sprague–Dawley rats, chronic liquid-diet exposure, and 24 h withdrawal. Withdrawal versus control showed directional agreement for all three original axes and their three deterministic derived indices, whereas chronic ethanol versus control agreed for only one of these six summaries. The derived indices do not constitute independent replication, and this two-study result is interpreted as contrast-specific directional concordance under similar protocols without identifying brain region as its cause. Across the broader alcohol-domain resource, effects occupied both directions and showed substantial between-contrast dispersion, defining a boundary to generalization rather than a universal alcohol response. The central interpretation was materially unchanged by alternative scoring algorithms, pathway catalogs, or cluster-balanced reanalysis of overlapping pathways; at a pathway-overlap (Jaccard similarity) threshold of 0.5, the latter preserved sample-level rankings (median Spearman ρ=0.983) and 76 of 78 pairwise effect directions. These results provide a reproducible framework for distinguishing local convergence from broader context dependence in rat alcohol transcriptomics. Candidate biomarkers should therefore be evaluated in harmonized exposure, withdrawal, region, sex, and species settings before translational interpretation.

## 1. Introduction

Alcohol use disorder (AUD) is widely recognized as a brain disorder involving distributed neural systems rather than a single pathological locus [1,2]. Core processes relevant to AUD, including reward valuation, motivational drive, contextual learning, emotional regulation, and stress adaptation, are mediated by partially overlapping but functionally distinct circuits such as the nucleus accumbens, ventral tegmental area, hippocampus, and amygdala [3,4]. This systems-level organization implies that ethanol exposure should not be interpreted as acting on one isolated substrate [5]. Instead, it is more plausibly viewed as a perturbation distributed across multiple neural contexts, where a common exposure may engage both shared and region-dependent molecular responses [6].

These four regions were included because the available rat datasets jointly sample complementary AUD-relevant functions. The nucleus accumbens and ventral tegmental area represent core mesolimbic reward circuitry [3,4], whereas the hippocampus contributes contextual learning and addiction-related memory [7], and the amygdala contributes affective regulation and withdrawal-related negative states [8,9]. Their inclusion was therefore determined by both biological relevance and the availability of region-resolved datasets with interpretable group structures; it was not intended to imply complete coverage of the neural circuitry of AUD.

Bulk transcriptomic studies have generated a substantial body of research on ethanol-related molecular changes in rodent brain tissues [10,11]. However, the field still lacks a coherent answer to a basic question: whether these studies collectively support a reproducible cross-region transcriptional pattern associated with ethanol exposure [12]. The difficulty is not simply that the datasets are numerous but that they are highly heterogeneous [13,14]. Existing studies differ in sampled brain region, exposure paradigm, withdrawal interval, assay platform, and statistical workflow [6,15]. They also often mix biologically distinct experimental settings, including acute exposure, chronic exposure, voluntary intake, forced administration, dependence-related models, and innate alcohol preference [16,17]. Because these conditions do not represent the same physiological or behavioral state, similarity across studies cannot be assumed, and discrepancy across studies cannot be dismissed as noise.

This leaves a central problem for the field. When studies are examined individually, they can describe local ethanol-responsive signatures within a particular region or paradigm, but they provide limited evidence about whether these signatures generalize across experiments [18,19]. When studies are combined, integration is often based on overlap among significant genes or pathways. Although useful, this strategy can obscure an important distinction: some inconsistency may reflect technical instability or low reproducibility [15], but some may instead reflect structured biological heterogeneity arising from region, model, or study design [20]. Without an explicit framework for separating these possibilities, pooled conclusions about “ethanol-responsive” transcription remain difficult to interpret.

The analysis was organized around two questions. First, do defined alcohol-related contrasts reproduce at the pathway level when the major experimental features are closely matched? Second, across the broader heterogeneous resource, how far does any local agreement generalize, and can the observed dispersion be attributed to recorded study features? The second question was treated as exploratory because region, paradigm, withdrawal interval, assay, and laboratory are sparse and confounded. We addressed these questions at the pathway level rather than by directly meta-analyzing gene-level ethanol coefficients. Each dataset was analyzed separately, projected onto the same predefined pathway axes, and represented by within-dataset standardized effects. This preserves the distinction among exposure, withdrawal, preference, and dependence-related states while allowing their directional patterns to be compared [9,21].

The framework comprises broad addiction-related, exercise-related, and stress-related pathway collections. Exercise and acute-stress datasets serve as positive-control contexts for the corresponding axes, not as alcohol cohorts. The exercise axis represents adaptive metabolic and plasticity-associated programs, whereas neuroinflammatory pathways are included directly within the addiction-related collection. This distinction is important because exercise has been investigated in alcohol use disorder and can alter ethanol seeking together with inflammatory and oligodendroglial responses [22,23], but it is neither an alcohol-specific mechanism nor a substitute for neuroimmune biology.

We first characterized within-dataset effects across four primary rat brain datasets. We then performed a focused sensitivity analysis in the two datasets with closely matched strain, sex, liquid-diet exposure, and 24 h withdrawal designs. Broader generalization was evaluated across additional alcohol-domain datasets using aligned contrast-level effects. Finally, we tested robustness to scoring algorithm, pathway-catalog breadth, and pathway redundancy and used bulk cell-marker enrichment as a secondary interpretive layer. This hierarchy separates direct within-dataset observations, matched-design replication, broad cross-study synthesis, and descriptive biological interpretation.

## 2. Results

### 2.1. A Cross-Region Transcriptomic Resource Captures Heterogeneous Ethanol-Responsive States

We assembled a primary rat brain transcriptomic resource spanning four anatomically and experimentally distinct datasets: hippocampus (GSE171051), ventral tegmental area (VTA; GSE233921), amygdala (GSE171400), and nucleus accumbens (NAc; GSE73627). Across these four primary datasets, 64 samples were retained after group assignment and preprocessing, including chronic ethanol, acute ethanol, withdrawal, voluntary ethanol, gavage, and matched control conditions (Supplementary Table S1). Because pathway scores were standardized within the dataset, inference focused on group contrasts and effect directions rather than absolute score levels across studies.

To project each sample into a shared biological space, we summarized pathway activity into three transcriptomic axes capturing addiction-related, exercise-related, and stress-related programs and then derived the addiction-dominant index (ADI), exercise-dominant index (ERI), and stress–activity balance (SAA). This framework compares disparate bulk transcriptomic studies in a common low-dimensional representation, but the scores remain relative to each dataset, and the three indices are deterministic functions of the original axes.

### 2.2. Primary Analyses Show Contrast-Dependent Pathway Reweighting

**Figure 1:**
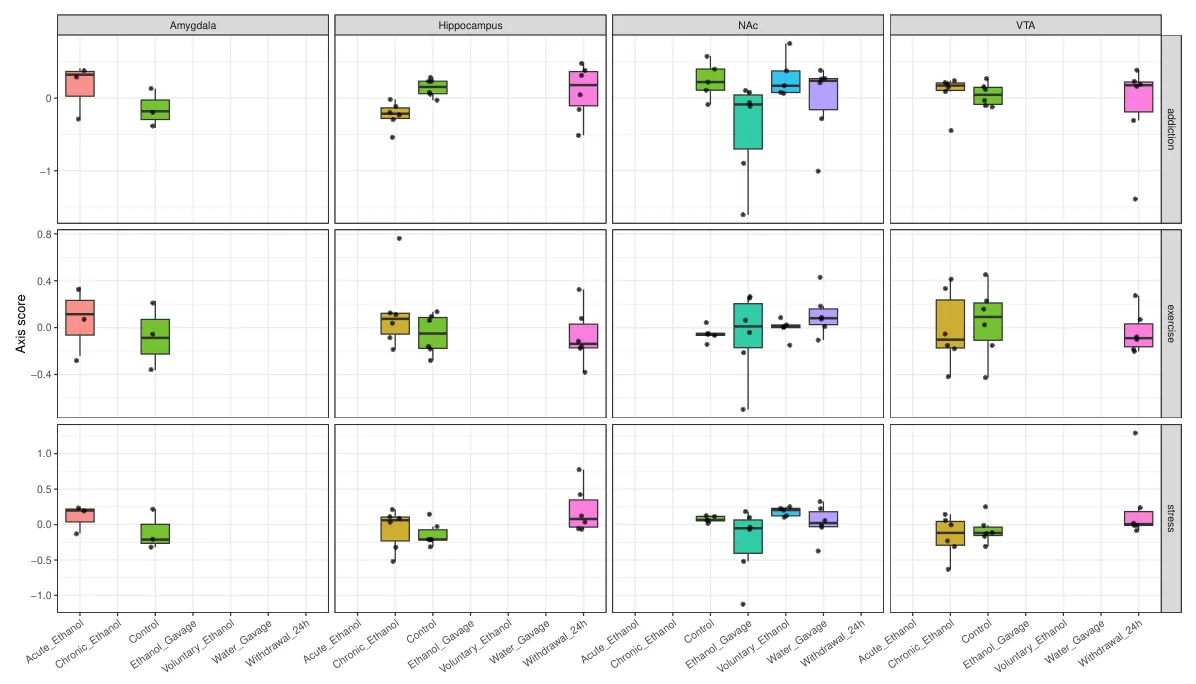
Cross-region distributions of the addiction, exercise, and stress transcriptomic axes. Boxplots show group-level distributions and overlaid points denote individual samples. For each pathway, ssGSEA scores were standardized across samples within the corresponding dataset before averaging. Zero therefore represents the dataset-specific mean relative enrichment; positive and negative values indicate relatively higher or lower enrichment, respectively. Blank x-axis positions indicate groups that were not present in the corresponding dataset. These values reflect relative enrichment of broad pathway collections, not the presence or absence of addiction-, exercise-, or stress-related activity.

**Figure 2:**
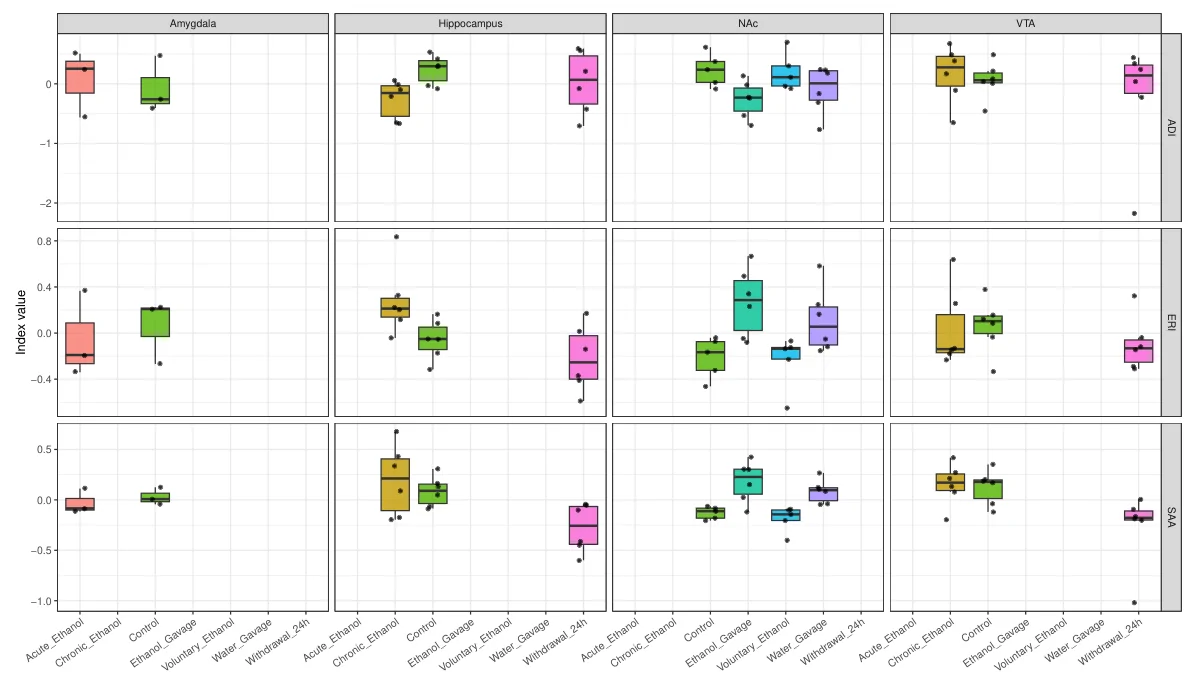
Cross-region distributions of the deterministic composite indices ADI, ERI, and SAA. Because the source axes were centered within the dataset, zero represents balance around the dataset-specific mean combination defined by each formula; positive and negative values indicate relative reweighting in opposite directions. Blank x-axis positions indicate groups that were not present in the corresponding dataset. The indices summarize selected pathway collections and should not be interpreted as diagnostic evidence that a biological program is present or absent.

Within-dataset contrasts differed in both magnitude and direction across the primary studies (Figure 1). The largest absolute addiction-axis effect occurred in the hippocampus for chronic ethanol versus control (|d|=2.43; Supplementary Table S3). In the same dataset, chronic ethanol and 24 h withdrawal occupied different positions in the axis/index space (Figure 2). In NAc, the largest shifts instead involved SAA and ERI contrasts among voluntary ethanol, ethanol gavage, and water-gavage groups. These observations identify strong within-dataset responses, but differences in region, exposure route, and comparison state prevent attribution to anatomy alone.

Composite indices captured additional group structure (Figure 2). In NAc, omnibus tests identified multi-group differences for SAA ($\mathrm{FDR}=5.98\times 10^{-3}$) and ERI ($\mathrm{FDR}=2.77\times 10^{-2}$; Supplementary Table S5). Hippocampal ERI and SAA also varied among control, chronic-ethanol, and withdrawal groups, whereas the VTA effects were smaller and the acute amygdala dataset had only three pooled RNA-seq samples per group. The primary result is therefore not a ranking of brain regions, but the observation that pathway reweighting depends on the specific exposure and reference contrast.

### 2.3. Matched Experimental Designs Reveal Contrast-Specific Directional Concordance

The hippocampal GSE171051 and VTA GSE233921 datasets provided the closest available design match: both used adult male Sprague–Dawley rats, a chronic ethanol liquid diet, a control-diet group, a 24 h withdrawal group, and six samples per group. This pairing allowed us to ask whether the same biological contrast reproduced after major design variables were held as similar as the public data permitted.

For 24 h withdrawal versus control, all three original axes and their three deterministic derived indices changed in the same direction in the two datasets, the rank correlation across the six effect-size summaries was 1.00, and the median absolute between-dataset difference in Cohen’s *d* was 0.215. Agreement weakened for withdrawal versus chronic ethanol: four of six summaries agreed in direction, the effect-size rank correlation was 0.486, and the median absolute difference was 0.867. For chronic ethanol versus control, only one of six summaries agreed in direction, the rank correlation was −0.371, and the median absolute difference was 1.165. Thus, withdrawal versus control showed complete directional concordance across the predefined summaries in these two closely matched studies, whereas the chronic-exposure contrast did not. The derived indices are mathematical functions of the original axes and therefore do not provide independent replication tests. Because the pair still differs in sampled region and unmeasured laboratory features, this analysis demonstrates contrast-dependent concordance but does not identify brain region as the causal source of disagreement. These results are summarized in Table 1.

### 2.4. Correlation Structure, Effect Sizes, and DEG Burden Define Dataset-Specific Response Profiles

**Figure 3:**
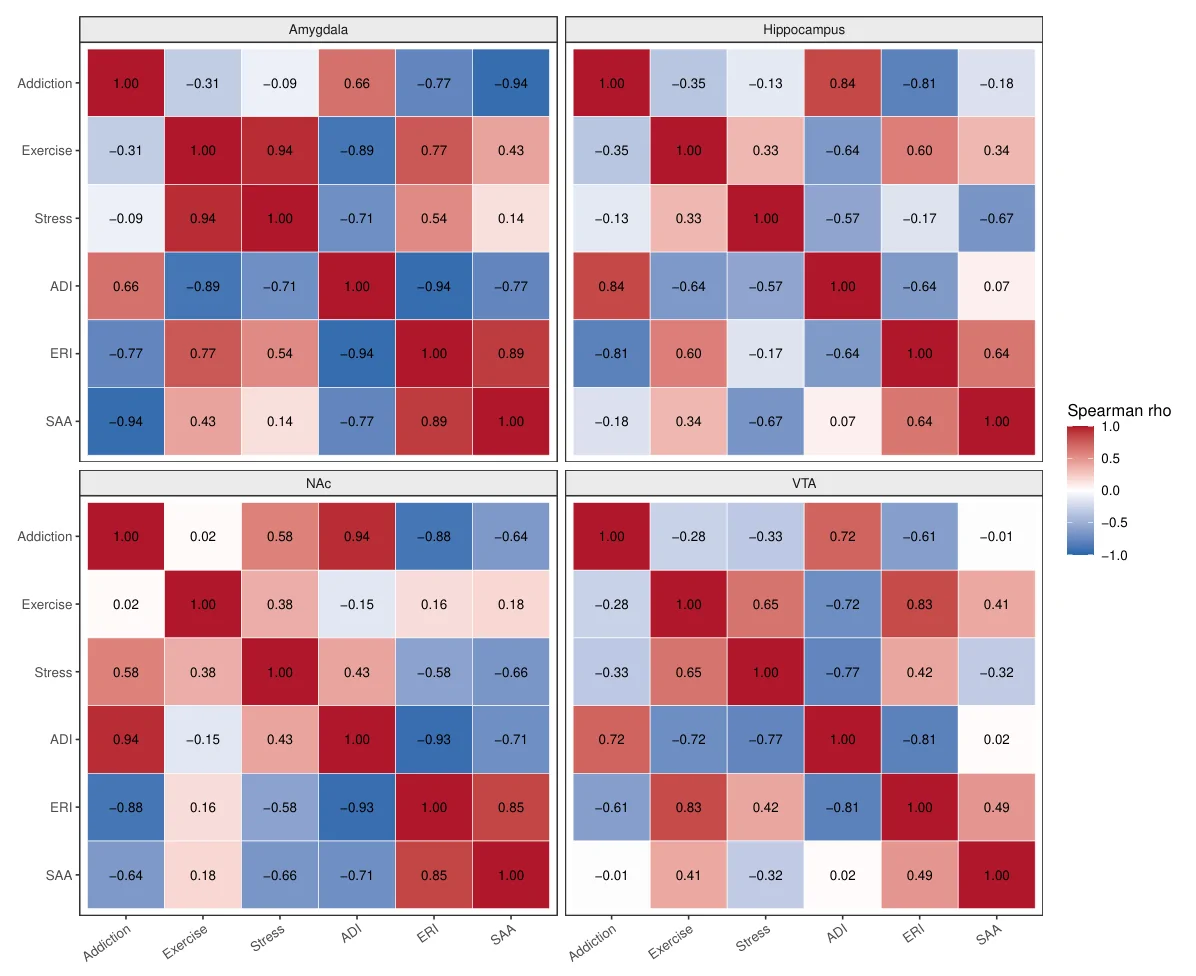
Within-dataset Spearman correlations among the addiction, exercise, and stress axes and the derived ADI, ERI, and SAA indices. Correlations among the three original axes provide the non-derived comparisons. Correlations involving ADI, ERI, or SAA are descriptive because these indices are deterministic linear combinations of the axes and their correlation structure is partly imposed by construction.

Correlations among the three original axes varied across datasets, with stronger exercise–stress alignment in the amygdala and VTA datasets than in the hippocampus and NAc datasets (Figure 3). Correlations involving ADI, ERI, and SAA are shown for descriptive completeness, but much of their strength is mathematically expected from the index formulas. We therefore do not treat those derived correlations as independent evidence of a region-specific transcriptomic architecture.

**Figure 4:**
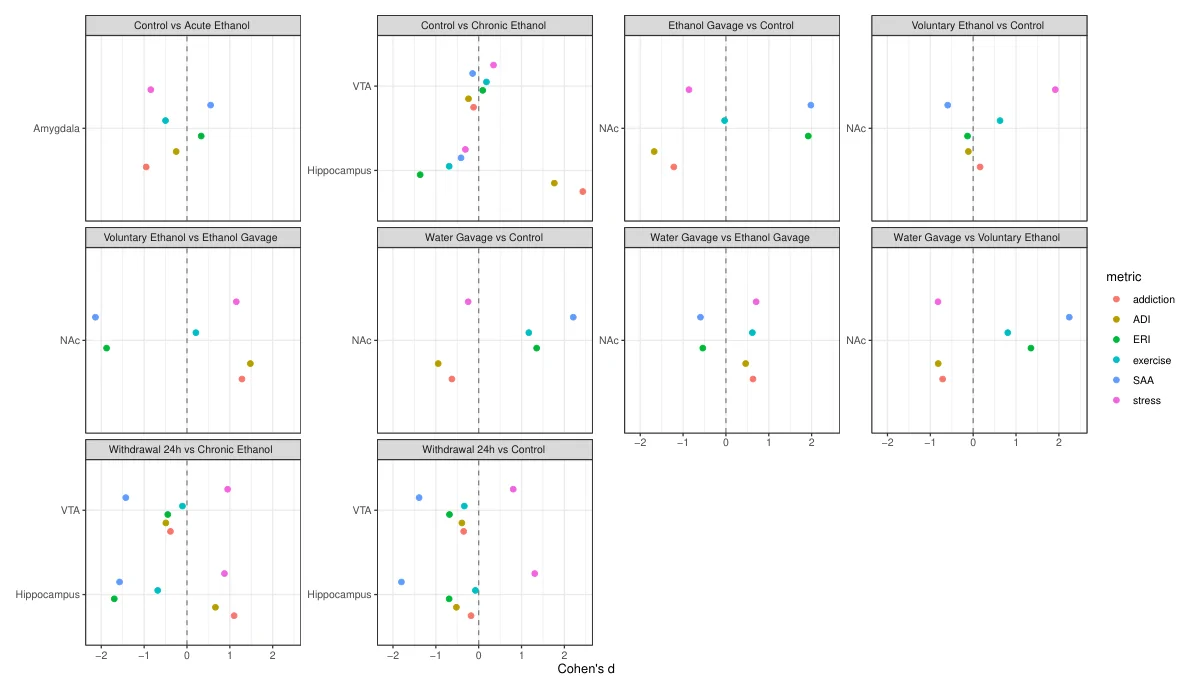
Forest-style summary of effect sizes across the four primary datasets. Points represent pairwise Cohen’s *d* values for each axis or index metric and each group comparison. Positive or negative values indicate the direction of the standardized group difference, with zero indicating no shift.

**Figure 5:**
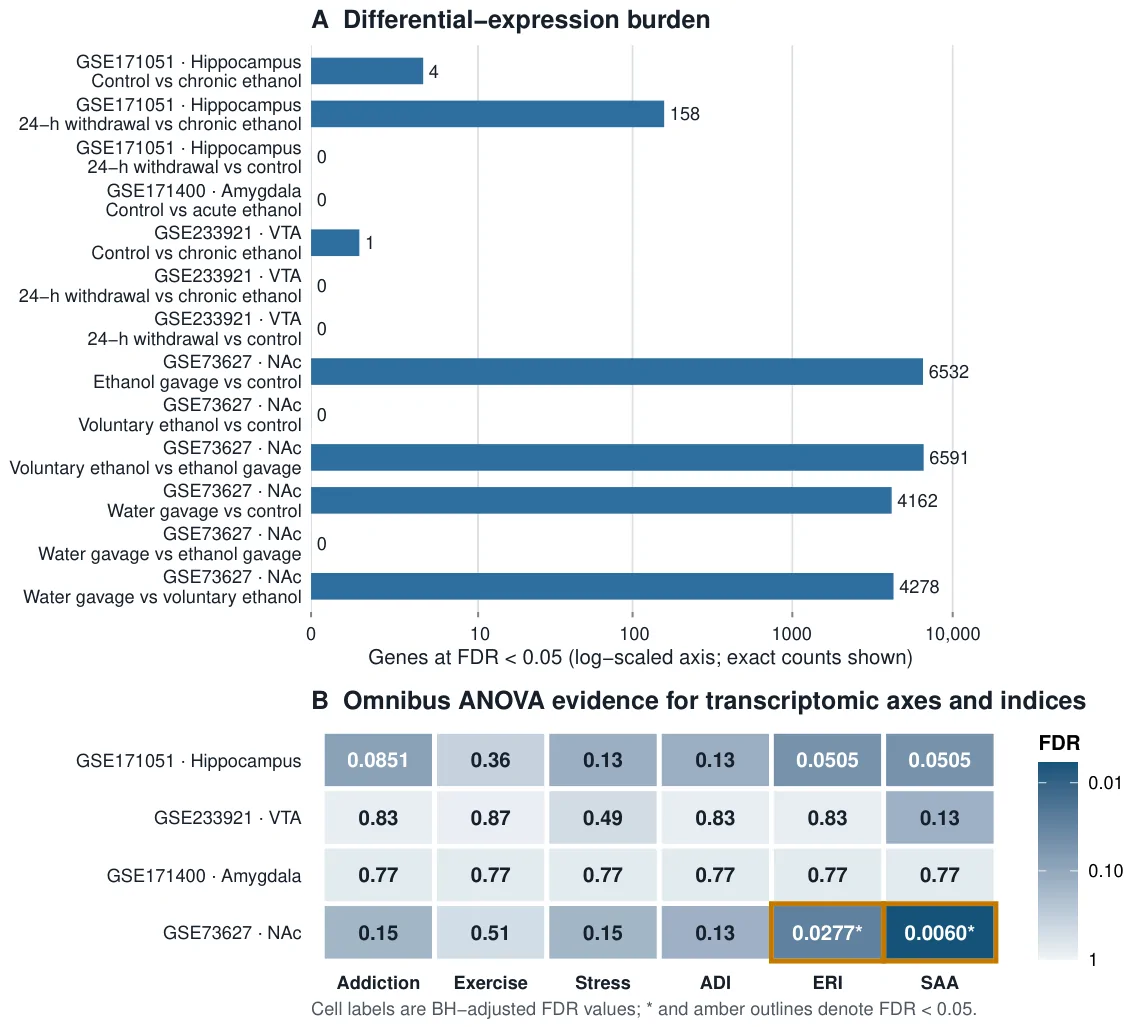
Readable summary of transcriptomic burden and omnibus statistics in the four primary datasets. Panel A shows the number of differentially expressed genes per comparison at a false-discovery rate (FDR) <0.05 on a log-scaled horizontal axis, with exact counts printed beside each bar. Panel B shows Benjamini–Hochberg-adjusted FDR values from omnibus ANOVA tests of the axis and composite-index metrics across groups within each dataset; asterisks and amber outlines denote FDR <0.05.

These differences were echoed in pairwise effect-size summaries (Figure 4). Several of the largest absolute effects in the full primary analysis involved NAc SAA and ERI comparisons, whereas the single strongest addiction-related displacement occurred in the hippocampus. Differential-expression summaries showed a related but not identical pattern (Figure 5). The highest transcriptomic burden was observed in NAc, where the voluntary ethanol versus ethanol gavage comparison yielded 6591 genes at FDR<0.05. By contrast, the hippocampal dataset was characterized less by extreme DEG counts than by larger standardized shifts along the addiction- and index-based axes.

Taken together, these analyses identify different response profiles across the primary datasets. NAc had the largest DEG burden and the strongest multi-group SAA/ERI contrasts, whereas the hippocampal dataset had the strongest addiction-axis displacement and a withdrawal group that remained distinct in the summarized transcriptomic coordinates. Correlation structure, effect sizes, and DEG burden were not maximized in the same dataset or metric. These are dataset-level observations; anatomical interpretation remains inseparable from differences in paradigm and laboratory context.

### 2.5. Cross-Dataset Synthesis Defines the Limits of a Universal Alcohol-Domain Shift

We next expanded the analysis beyond the four primary datasets by incorporating eight additional datasets spanning alcohol-risk, alcohol-dependence, binge-ethanol, innate-preference, exercise, and acute-stress paradigms. These external datasets were used as comparative cohorts and as calibration material for directional behavior of the axis framework. Directional checks across pre-specified expectations showed that 7 of 10 control contrasts moved in the expected direction, including both adolescent binge-ethanol datasets (GSE49040 and GSE49041) and the exercise and acute-stress reference datasets. The one-sided exact binomial result was inconclusive (P=0.172); accordingly, this analysis was used only as a calibration check and not as evidence that the axes are alcohol-specific or externally validated.

**Figure 6:**
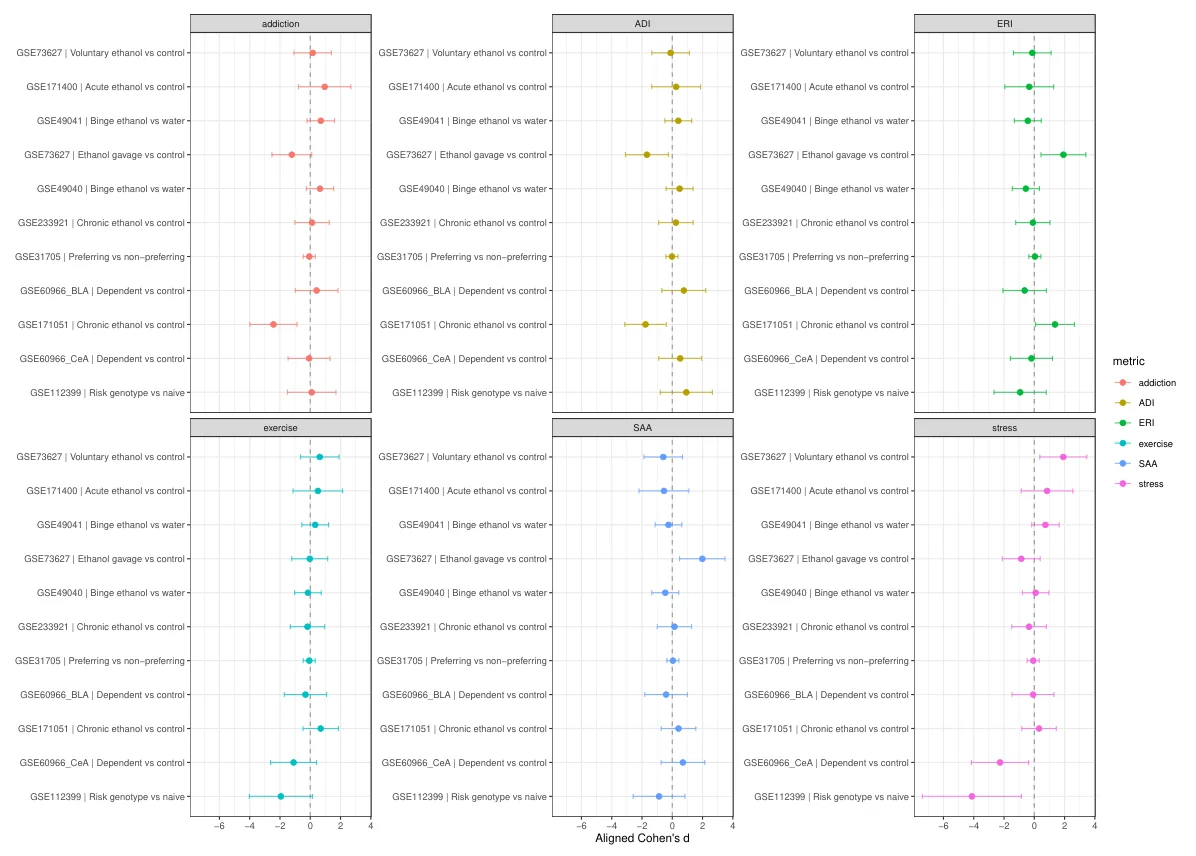
Aligned alcohol-domain effect sizes across primary and external datasets. For each contrast-level estimate, Cohen’s *d* was oriented so that positive values indicate higher scores in the alcohol-exposed, alcohol-dependent, alcohol-preferring, or alcohol-risk group relative to its matched reference group. Error bars indicate approximate 95% confidence intervals. Group sample sizes for the primary datasets are reported in Supplementary Table S1; pooled meta-analysis results are reported in Supplementary Table S6.

Given the biological and technical diversity of the broader resource, interpretation was anchored in random-effects summaries, with fixed-effect estimates retained as complementary precision-weighted descriptions (Figure 6; Supplementary Table S6). Across 11 alcohol-domain contrasts, the addiction metric showed effects in both directions and a near-zero random-effects mean ($d_{\mathrm{RE}}=0.007$, where RE denotes random effects; 95% CI −0.447 to 0.460); stress and addiction showed appreciable between-contrast heterogeneity ($I^{2}=58\%$ and 46%, respectively). Similar dispersion characterized ADI, ERI, SAA, and exercise. Leave-one-dataset-out analysis did not convert this distributed pattern into a stable universal shift (Supplementary Table S16). Because some contrasts share a dataset or parent study, these models summarize the observed contrast distribution rather than independent replications. The cross-dataset result is therefore the coexistence of locally concordant contrasts and broad context-dependent divergence, not a single alcohol-domain score direction.

For transparency, we also screened six metrics against four recorded study-feature families (24 secondary omnibus tests). None met the Benjamini–Hochberg-adjusted threshold; the smallest raw value was the region-family test for the addiction metric (unadjusted P=0.042, q=0.418; Supplementary Table S20). Because the feature levels are sparse and confounded with paradigm, assay, and laboratory, this screen was not used to define the title, abstract, or primary interpretation. Positive-control recovery and bulk cell-marker associations were likewise retained as calibration and interpretation layers rather than evidence of alcohol specificity.

### 2.6. The Conclusions Are Robust to Scoring Method, Pathway Catalog, and Pathway Redundancy

**Figure 7:**
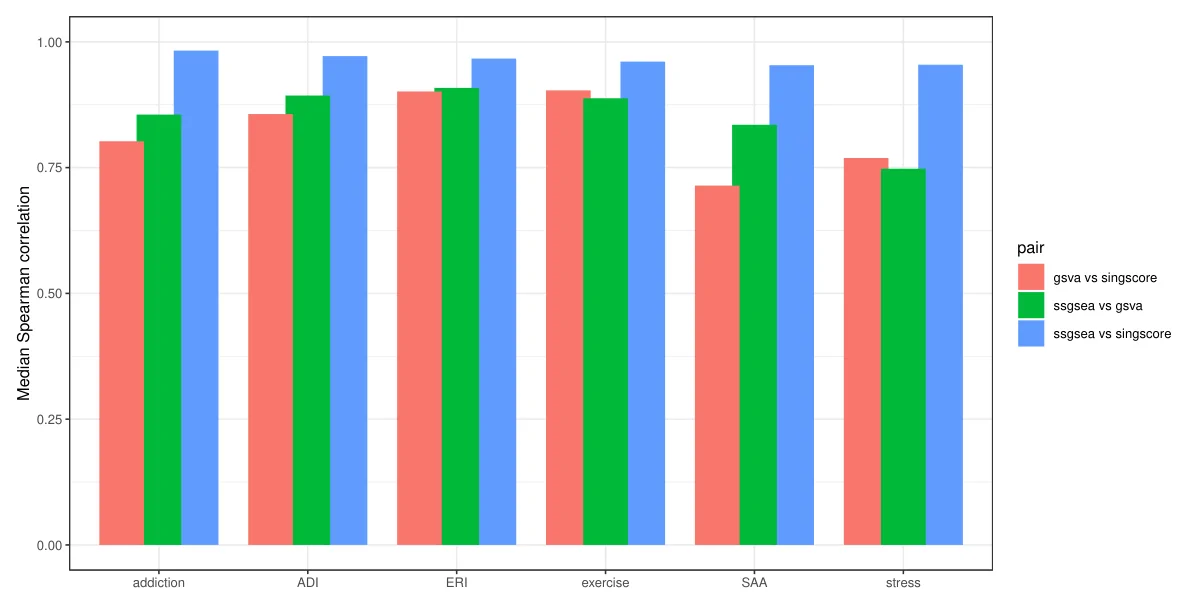
Median sample-level Spearman correlations between ssGSEA-, GSVA-, and singscore-derived axis or composite-index values across the four primary datasets. This figure evaluates whether the main conclusions depend strongly on the pathway-scoring method.

We then asked whether the conclusions depended strongly on the analytical implementation. Re-scoring the four primary datasets with GSVA and singscore showed high concordance with the ssGSEA implementation, particularly between ssGSEA and singscore, for which median sample-level Spearman correlations were approximately 0.95–0.98 across the axes and indices (Figure 7). GSVA concordance was lower for several metrics, but the principal within-dataset contrast patterns were retained.

**Figure 8:**
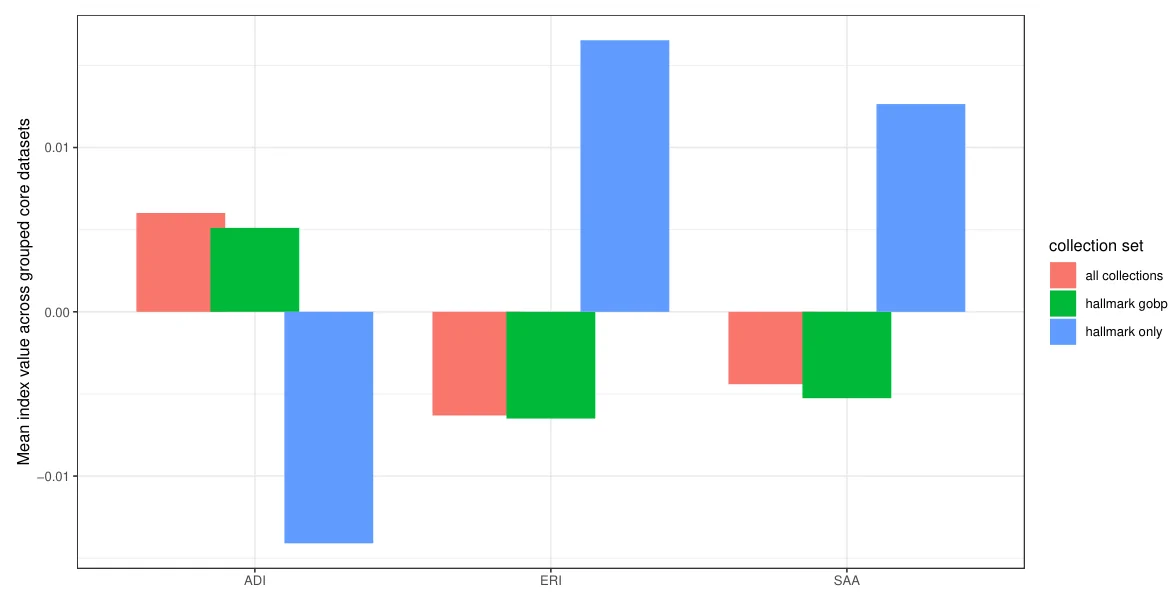
Grouped mean index values after recomputing the primary datasets with pathway collections of different breadth, including hallmark-only, hallmark plus GO biological process, and broader combined collections. This figure evaluates sensitivity of the qualitative index behavior to pathway-catalog definition.

Recalculation with Hallmark-only, Hallmark plus GO biological process, and broader combined catalogs likewise retained the qualitative contrast behavior (Figure 8). We then directly addressed correlated gene-set weighting by collapsing pathways into overlap clusters and assigning equal weight to each cluster. At the prespecified Jaccard threshold of 0.5, the cluster-balanced axes retained a median sample-level correlation of 0.983 with the original scores (minimum 0.829) and preserved 76 of 78 pairwise effect directions. Across Jaccard thresholds of 0.3–0.7, median correlations ranged from 0.959 to 0.997 and direction preservation ranged from 89.7% to 98.7% (Supplementary Tables S24–S25; Supplementary Figure S7). Thus, the central contrast-dependent result was not created by repeated weighting of a densely overlapping pathway family.

The complete keyword rules are provided in Supplementary Table S11, and the selected candidate pathway registry is provided in Supplementary Table S12. A matched random-pathway negative control exceeded random sets in some, but not all, primary settings (Supplementary Table S13), which is why individual axis labels are interpreted as broad program summaries rather than specific biomarkers. Supplementary Table S14 grades the evidential strength and boundary conditions of each main conclusion.

### 2.7. Cell-Type Marker Analysis Provides a Supportive Bulk-Level Interpretive Layer

**Figure 9:**
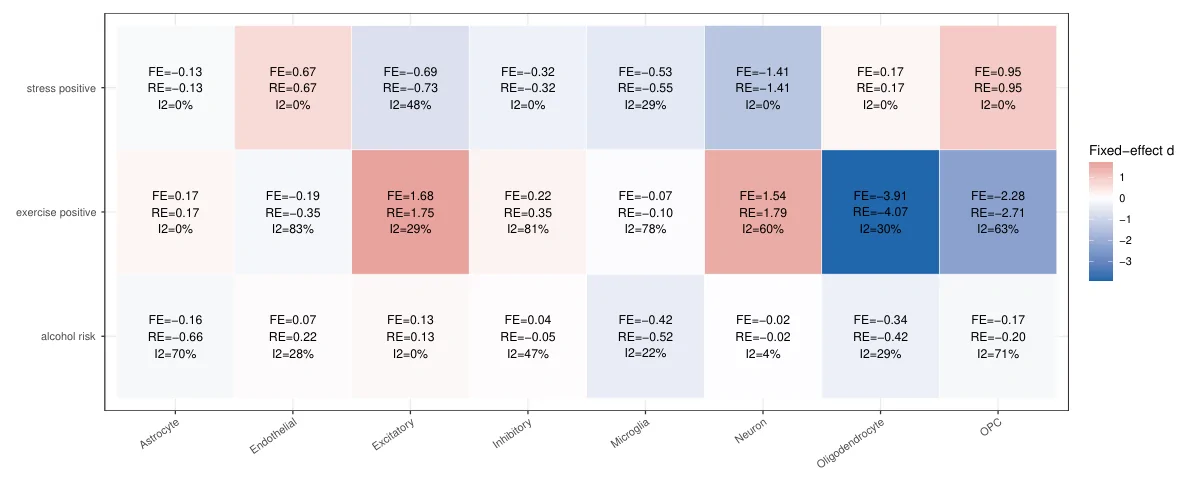
Precision-weighted fixed-effect pooled marker-enrichment shifts for canonical neuronal, glial, vascular, and precursor populations across the alcohol-risk, exercise-positive, and stress-positive domains. Tile labels report fixed-effect (FE) and random-effects (RE) pooled estimates together with $I^{2}$. Marker scores were computed as average standardized expression across detected marker genes and should be interpreted as relative bulk marker enrichment. OPC denotes oligodendrocyte precursor cell. Cell-type leave-one-dataset-out sensitivity results are reported in Supplementary Table S18.

As a secondary biological interpretation layer, we quantified canonical neuronal, glial, vascular, and precursor marker-enrichment scores. Across the 11 alcohol-domain contrasts, microglial marker enrichment showed the most consistent summary shift ($d_{\mathrm{RE}}=-0.52$, 95% CI −0.89 to −0.15), followed by oligodendrocyte markers ($d_{\mathrm{RE}}=-0.42$, 95% CI −0.81 to −0.03; Figure 9; Supplementary Table S9). Leave-one-dataset-out analysis retained the negative microglial summary, whereas the oligodendrocyte result was more sensitive (Supplementary Table S18). Assay- and quality-stratified differences further argue against a universal cellular interpretation (Supplementary Table S21). These values are relative bulk marker summaries, not cell fractions or mechanistic validation. Correlations between marker scores and the axes are reported only as descriptive context in Supplementary Figure S6.

### 2.8. Dataset Inclusion and Quality Grading Define the Boundaries of Interpretation

We also applied the predeclared quality rubric in Supplementary Table S10. The primary and positive-control datasets carried the strongest interpretive weight, whereas external alcohol-domain comparison datasets mainly defined generalization boundaries. GSE31705 was Tier C because it lacked a directly matched exposure-control design, and GSE159136 was excluded because no matched control could be recovered.

## 3. Discussion

The central result is that directional concordance in rat alcohol transcriptomics depends on the biological contrast being compared. This conclusion is strongest in the two closest protocol-matched datasets. Their 24 h withdrawal-versus-control effects agreed in direction for all three original axes and their three deterministic derived indices and had identical rank ordering, whereas their chronic-ethanol-versus-control effects agreed for only one of these six summaries. Because the derived indices are mathematical functions of the original axes, they are not counted as independent replications. The comparison therefore provides a focused two-study concordance result under similar strain, sex, exposure, withdrawal, and group-size conditions while also showing that protocol similarity does not guarantee a universal chronic-exposure signature. Because brain region and laboratory remain coupled in this pair, the result supports contrast-specific concordance rather than an independent anatomical effect.

The remaining primary datasets broaden this picture. NAc had the largest differential-expression burden and clear multi-group SAA/ERI structure, whereas hippocampus had the largest addiction-axis displacement. These observations are compatible with context-dependent weighting of partially shared pathway programs [5,6,24], but they cannot be attributed to anatomy alone because region, exposure route, time point, assay, and laboratory differ together. Correlations among the original axes provide descriptive support for dataset-specific organization; correlations involving ADI, ERI, and SAA are partly predetermined by their formulas and were not treated as independent evidence.

Three sensitivity analyses reduce the likelihood that this conclusion is an artifact of pathway scoring. The principal contrast patterns were retained with alternative enrichment algorithms and catalogs of different breadth. More importantly, cluster balancing explicitly prevented a large family of overlapping gene sets from receiving disproportionate weight. At a Jaccard threshold of 0.5, this analysis preserved 76 of 78 effect directions and produced a median sample-level correlation of 0.983 with the original scores; comparable results were obtained across thresholds. Pathway overlap therefore affects the exact numerical scale but does not explain the central distinction between reproducible withdrawal contrasts and divergent chronic-exposure contrasts.

The broader synthesis tests a different question: whether diverse alcohol-related paradigms share one direction after their contrast-level effects are aligned. They did not. Effects were distributed in both directions, heterogeneity was appreciable, and leave-one-dataset-out analysis did not recover a stable universal shift. This does not negate the strong local effects or the matched withdrawal replication. Instead, it defines their boundary: a signal can be reproducible for a specified exposure state without being a universal alcohol-domain endpoint [15]. The secondary study-feature screen was retained only as a transparent accounting of possible sources of dispersion; its sparse, confounded categories cannot adjudicate among region, paradigm, assay, and laboratory.

The cell-marker layer adds biological context but not direct biological validation. Microglial and oligodendrocyte marker summaries were directionally aligned across fixed- and random-effects models [25,26], but their magnitudes varied by assay and quality stratum. These bulk marker-enrichment summaries neither deconvolve cell fractions nor establish cell-state mechanisms, causal direction, or tissue-composition changes.

### Limitations

Several limitations define the scope of inference. First, this is a purpose-built public-data resource rather than a prospectively registered systematic review. A revision-stage electronic audit documents reproducible queries and record counts, but the original record-level screening history was not retained; the resource is therefore not exhaustive or PRISMA-complete. Second, region, paradigm, strain, assay, time point, and laboratory are extensively confounded. The matched-design analysis reduces several differences but contains only two datasets and cannot estimate an independent brain-region effect. Third, all primary datasets used adult male rats. Human brain tissue is difficult to obtain in well-controlled exposure and withdrawal states, but the absence of human validation remains a limitation rather than a justification for clinical extrapolation. Rodent paradigms do not reproduce the behavioral, social, or clinical complexity of human AUD, and the results require testing in females, other species, additional regions, and human tissue. Fourth, transcriptomic changes can be modest relative to technical variation and bulk-tissue composition. Bulk RNA measurements average cell states and cell proportions; marker enrichment is descriptive and is neither deconvolution nor evidence of a causal cellular mechanism [27]. Fifth, the pathway collections are broad and not disease-specific. Cluster balancing reduces concern about repeated weighting of overlapping gene sets, but it cannot make the labels unique or diagnostic. Sixth, ADI, ERI, and SAA are deterministic combinations of the original axes, so their correlations are not independent evidence. Finally, fixed- and random-effects models summarize aligned contrast-level effects under marked heterogeneity. Some contrasts share a GEO series or parent study, and conventional models do not fully represent that dependence. Accordingly, pooled values and leave-one-dataset-out analyses characterize directional stability and dispersion; they do not estimate a universal individual-level alcohol response.

The next biological tests should use harmonized exposure and withdrawal designs, both sexes, additional regions and species, and single-cell or spatial measurements. Such studies could distinguish cell-state transitions from compositional changes and separate ethanol effects from handling or paradigm effects more directly than retrospective integration can.

## 4. Materials and Methods

### 4.1. Study Design, Dataset Curation, and Analysis Overview

Publicly available rat brain transcriptomic datasets were obtained from the Gene Expression Omnibus (GEO; https://www.ncbi.nlm.nih.gov/geo/ (accessed on 15 May 2026)) [28]; GEO Series (GSE) accession numbers are used throughout. This study uses a purpose-built analytical resource rather than an exhaustive systematic-review sample. Dataset discovery combined targeted GEO searches using rat, brain-region, alcohol/ethanol, exposure, withdrawal, dependence, preference, and transcriptomic terms with accession and citation tracing. Eligibility required region-resolved bulk expression data, a biologically interpretable contrast, recoverable sample annotations, and sufficient within-group replication for a contrast-level effect estimate. Twelve datasets were retained and assigned before synthesis to primary, external alcohol-domain comparison, or exercise/stress positive-control roles. One additional series was inspected in depth but excluded because a matched control could not be recovered.

To make the electronic discovery step transparent, we also performed a revision-stage GEO audit on 17 August 2026. A broad series-level query combining GSE[ETYP], Rattus norvegicus[Organism], brain-region terms, alcohol/ethanol terms, and expression-profiling dataset types returned 137 records; a title-field refinement returned 82 records. The documented query components, audit date, and record counts are reported in the Table S10 Search Audit worksheet of the Supplementary Tables file. These audit counts document the sensitivity of the electronic search but are not presented as retrospective Preferred Reporting Items for Systematic Reviews and Meta-Analyses (PRISMA) flow counts because the original assembly was not prospectively registered and did not retain record-level screening assignments. The accession and analytical-role registry was checked during revision by the authors responsible for formal analysis (H.A. and Z.W.); no independent dual-screening agreement statistic is claimed. The complete registry reports each included accession, analytical role, comparison structure, sample size, quality tier, and the in-depth exclusion reason. We therefore do not claim exhaustive coverage of all rat alcohol transcriptomic studies.

The primary analysis set comprised hippocampus (GSE171051 [29]), ventral tegmental area (VTA; GSE233921 [30]), amygdala (GSE171400 [8]), and nucleus accumbens (NAc; GSE73627 [31]). Table 2 distinguishes their exposure mode, collection window, animal characteristics, and analyzed group structure. These datasets are biologically informative but not exchangeable: they include chronic liquid-diet exposure, 24 h withdrawal, acute intraperitoneal exposure, voluntary drinking, and intragastric gavage. The primary analysis therefore estimated effects within each dataset and did not pool individual animals across studies.

External alcohol-domain comparison datasets included GSE112399 [32] (NAc alcohol-risk genotype); GSE49040 [33] (central amygdala adolescent binge ethanol); GSE49041 [33] (NAc shell adolescent binge ethanol); GSE60966_BLA [34], representing basolateral amygdala (BLA) alcohol dependence; GSE60966_CeA [34], representing central amygdala (CeA) alcohol dependence; and GSE31705 [35], representing the nucleus accumbens shell component of the broader extended-amygdala study (PubMed identifier 24157127). Positive-control datasets included GSE299436 [36] (hippocampal exercise) and GSE126705 [37] (hippocampal acute stress). The processed CeA count matrix GSE159136 [38] was screened but excluded from alcohol-domain comparison because a matched control group could not be recovered with sufficient confidence from the processed files.

**Figure 10:**
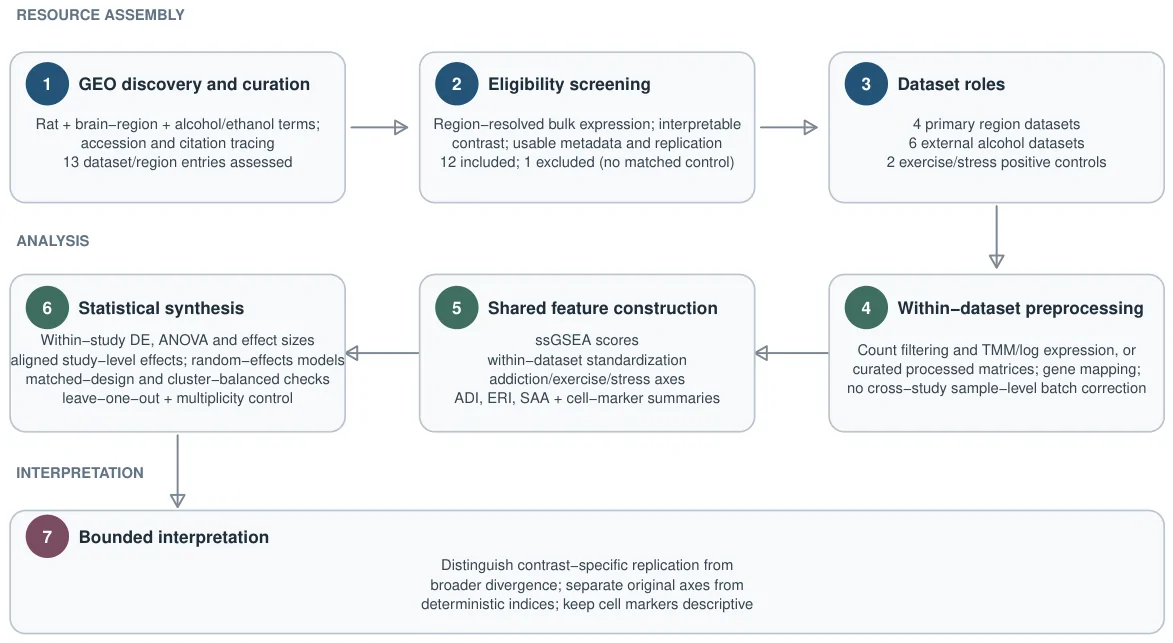
Reproducible analysis workflow. GEO candidates were screened using predefined biological and metadata criteria, assigned to primary, external alcohol-domain comparison, or positive-control roles, and processed within the dataset. Pathway scores and composite indices were calculated at sample level, whereas cross-study synthesis used aligned contrast-level effects. The evidential hierarchy distinguishes within-dataset contrasts, a focused matched-design comparison, broader cross-study synthesis, scoring and pathway-redundancy sensitivity analyses, and descriptive cell-marker interpretation. Abbreviations: TMM, trimmed mean of M values; ssGSEA, single-sample gene set enrichment analysis; ADI, addiction-dominant index; ERI, exercise-dominant index; SAA, stress–activity balance; DE, differential expression; ANOVA, analysis of variance.

Datasets were classified using a transparent rubric that considered assay resolution, presence of an interpretable reference group, total sample size, minimum within-group replication, and regional annotation clarity. This framework was used to distinguish datasets that primarily supported the main biological claims from those that mainly informed generalizability and boundary conditions. The complete analysis workflow is summarized in Figure 10.

Table 2 also makes the biological-state differences explicit. GSE171051 and GSE233921 contain chronic liquid-diet exposure and 24 h withdrawal groups and provide the dependence-related matched comparison; GSE171400 is an acute, non-withdrawal exposure study; and GSE73627 contains two voluntary/gavage experiments rather than one simple alcohol-versus-control contrast. Sex and adult status are reported for every primary dataset, exact age is reported where recoverable, and no inference was based on an unreported age, dose, time point, or behavioral phenotype.

The analysis was restricted to rats because the original resource-building objective was a region-resolved synthesis within one species, and the available rat series provided complementary reward, affective, memory, withdrawal, and exposure paradigms with recoverable sample-level data. This choice should not be interpreted as evidence that rat datasets are more numerous or intrinsically more generalizable than mouse datasets. A mouse extension would increase the candidate pool but would also introduce a species boundary and require a separately harmonized design; it is identified as a priority for future cross-species validation.

### 4.2. Expression Preprocessing and Gene Harmonization

Raw-count datasets were downloaded from GEO supplementary files and imported as sample-by-gene-count matrices. For datasets distributed as per-sample featureCounts-style outputs [39], gene-level counts were aggregated across files to build a combined matrix. Genes were filtered at the count level using a counts-per-million threshold and normalized using the trimmed mean of M values (TMM) procedure [40] implemented in edgeR [41]. Log-expression values were then computed from normalized counts.

To allow cross-dataset comparison, gene identifiers were mapped to rat gene symbols using org.Rn.eg.db [42] through the AnnotationDbi framework [43,44]. Entrez-style and Ensembl-style identifiers were converted to symbols, and duplicated symbols were collapsed by summation for count-derived matrices or by mean expression for processed matrices. For array-based or matrix-style external comparison datasets, expression matrices were first restricted to annotated gene-symbol rows, collapsed to unique symbols, and then passed into the same pathway-scoring framework used for the primary datasets.

Sample-group assignments were derived from accession-specific sample metadata, GEO phenotype tables, or sample-name patterns depending on the structure of each dataset. Samples with unresolved labels were excluded before analysis. No missing-value imputation was performed, and no sample-level outlier removal was applied beyond preprocessing filters and metadata-based exclusion. Because study-specific batch variables were not consistently recoverable across GEO series, no cross-study batch correction was attempted; instead, all inferential models were fit within the dataset, and cross-study synthesis was performed only on contrast-level effect sizes. Primary-dataset group counts, inclusion decisions, and dataset-level model specifications are consolidated in the Supplementary Tables file (worksheets Tables S1, S10, and S19).

### 4.3. Primary Pathway-Axis and Composite-Index Framework

Rat-mapped Molecular Signatures Database (MSigDB) pathway collections were used to define addiction-related, exercise-related, and stress-related pathway sets [45,46]. Pathways were selected from Hallmark, Gene Ontology biological process (GO:BP), and Reactome collections [47,48,49] by keyword matching to prespecified terms. The addiction axis deliberately included reward, dopaminergic, synaptic, glutamatergic, gamma-aminobutyric acid (GABA)-related, microglial, inflammatory, cytokine, and complement terms; neuroinflammatory programs were therefore represented directly rather than replaced by the exercise component. Exercise-axis terms emphasized mitochondrial, oxidative-phosphorylation, metabolic, neurotrophic, angiogenic, vascular, recovery, and anti-inflammatory programs, whereas the stress axis emphasized glucocorticoid, oxidative, endoplasmic-reticulum, heat-shock, autophagy, and deoxyribonucleic acid (DNA) damage responses. These labels describe broad pathway collections and should not be interpreted as disease-specific biomarkers. After gene filtering and symbol harmonization, each pathway was intersected with the corresponding expression matrix, and sets with fewer than 10 detected genes were discarded.

To increase pathway-selection transparency, complete keyword and matching rules are provided in Supplementary Table S11, and the selected candidate pathway registry is provided in Supplementary Table S12. We additionally quantified redundancy after dataset-specific expression filtering. Across the four primary datasets, one gene appeared in an average of 3.06–4.25 retained pathways within an axis, with maxima of 36–44 memberships. Median pairwise pathway Jaccard similarity, defined as intersection size divided by union size, was zero and the 90th percentile was 0.017–0.034, indicating that most pairs had little overlap; however, the maximum similarity was 0.846–0.923, demonstrating a small highly redundant subset. Cross-axis unique-gene Jaccard similarity ranged from 0.202 to 0.273 (Supplementary Table S22). These results show that averaging reduces reliance on any single pathway but does not eliminate repeated gene weighting. As a separate framework-level negative control, random pathway sets were sampled from the same collections after excluding keyword-matched pathways (Supplementary Table S13).

Single-sample gene set enrichment analysis (ssGSEA) was the primary scoring method [50]. For each sample, genes were ranked by expression; for each pathway, ssGSEA contrasted the empirical cumulative distributions of genes inside and outside that set and integrated the rank-based difference to obtain a sample-specific enrichment score. Scores for each pathway were then standardized across samples within the same dataset (mean 0, standard deviation 1) and averaged within each axis. Thus, an axis value of zero denotes the dataset-specific mean relative enrichment of the selected pathway collection, and positive or negative values denote relatively higher or lower enrichment within that dataset. The values do not indicate the presence or absence of addiction, exercise, or stress biology and are not directly comparable as absolute activity across datasets.

From these three axis scores, we defined the addiction-dominant index (ADI), exercise-dominant index (ERI), and stress–activity balance (SAA) as follows:

ADI=addiction−0.5(exercise+stress),

ERI=exercise−0.5(addiction+stress),

SAA=exercise−stress.

ADI captured addiction-axis reweighting relative to the other two programs, ERI captured exercise-axis reweighting, and SAA summarized the exercise-minus-stress contrast. These indices are deterministic linear combinations of the original axes and were treated as descriptive summaries rather than independent biological measurements.

### 4.4. Primary Statistical Analyses of Within-Dataset Transcriptomic Reweighting

Primary analyses combined differential-expression testing, omnibus group comparisons for axes and composite indices, and pairwise effect-size estimation. For datasets with raw counts, differential expression was tested in edgeR [41] using a design matrix with group as the only modeled term, reflecting the limited availability of harmonized within-study covariates across GEO series. Genes meeting false-discovery rate (FDR) thresholds after Benjamini–Hochberg (BH) correction [51] were classified as differentially expressed genes (DEGs) and summarized per comparison as a measure of transcriptomic burden. For axis and composite-index metrics, omnibus group structure was evaluated with one-way analysis of variance (ANOVA) using the model metric∼group, and pairwise standardized differences were summarized with Cohen’s *d* [52] together with approximate 95% confidence intervals (CIs) derived from standardized mean-difference variance estimates [53]. Group sample sizes for the primary datasets are reported in Supplementary Table S1, and dataset-level sample-size summaries are reported in Supplementary Table S10.

Within-region correlation structure among addiction, exercise, stress, ADI, ERI, and SAA was summarized using sample-level Spearman correlation matrices [54] computed separately for each primary dataset. Correlations among the three original axes were evaluated as the non-derived relationships of interest. Correlations involving ADI, ERI, or SAA were retained only as descriptive checks because their formulas impose part of the observed correlation structure; they were not treated as independent evidence of regional architecture.

Multiple-testing control was applied within predefined analysis families. Benjamini–Hochberg adjustment was applied separately within each differential-expression contrast, within each dataset-level family of omnibus axis/index tests, and within each dataset-level family of cell-type analysis-of-variance tests. A secondary study-feature screen comprised 24 tests (six metrics by four recorded feature families); its omnibus *P* values were adjusted together by the Benjamini–Hochberg procedure, with raw and adjusted values reported in Supplementary Table S20. This screen was not used to define the title, abstract, or primary interpretation. Subgroup synthesis, leave-one-dataset-out analyses, and exact direction checks were treated as sensitivity analyses. Unless otherwise stated, sample-level models were fit only within dataset, and cross-dataset synthesis used contrast-level standardized effects.

### 4.5. Robustness Analyses of Scoring Method, Pathway Definition, and Pathway Redundancy

Robustness of the axis framework was evaluated at three analytical levels. First, the four primary datasets were rescored using gene set variation analysis (GSVA) [55] and singscore [56] in addition to ssGSEA [50]; concordance was summarized by sample-level Spearman correlation. Second, the analyses were repeated with pathway catalogs of different breadth, including Hallmark-only, Hallmark plus GO biological process, and a broader combined collection. Third, we tested whether nested or gene-overlapping pathways disproportionately determined the axes. Within each dataset and axis, pathways were connected when their gene-set Jaccard similarity exceeded 0.3, 0.5, or 0.7. Connected components were treated as redundancy clusters. Standardized pathway scores were averaged within each cluster and then averaged across clusters so that a dense family of overlapping pathways received the same total weight as a singleton pathway. Sample-level rankings and all within-dataset pairwise effect directions were compared between the original and cluster-balanced axes. Detailed results are reported in Supplementary Tables S24–S25 and summarized in Supplementary Figure S7.

Pre-specified expected-direction checks were also applied to external alcohol-domain comparison datasets and to exercise and acute-stress positive-control datasets. These checks assessed directional behavior outside the primary analysis set but were not treated as independent biological validation. The match count was accompanied by an exact one-sided binomial test against a null success probability of 0.5. None of the robustness analyses were used to redefine the pathway collections or primary contrasts after inspecting the results.

### 4.6. Focused Matched-Design Sensitivity Analysis

To reduce the design heterogeneity emphasized by the reviewers, we identified the closest protocol-matched comparison available in the primary resource. GSE171051 and GSE233921 both used adult male Sprague–Dawley rats, chronic ethanol liquid-diet exposure, control-diet groups, and 24 h withdrawal groups, with six samples per group. For each of the three original axes and three deterministic derived indices, Cohen’s *d* was oriented identically for chronic ethanol versus control, 24 h withdrawal versus control, and 24 h withdrawal versus chronic ethanol. We then compared effect direction, rank ordering across the six summaries, and absolute between-region differences. Because the derived indices are functions of the original axes, concordance across the six summaries was not interpreted as six independent replication tests. This focused analysis tests whether heterogeneity persists after major design variables are held as similar as the public data allow; with only two datasets, it does not estimate an independent region effect. Results are reported in Supplementary Tables S23 and S23B and Supplementary Figure S7.

### 4.7. External Alcohol-Domain Comparisons, Aligned Contrast Registry, and Cross-Dataset Synthesis

To synthesize information across primary and external datasets, we specified an aligned contrast registry in which each comparison was oriented relative to a biologically interpretable low-reference and high-perturbation group. Standardized mean differences were then aligned so that the sign of each effect had a consistent interpretation within each biological domain. This registry was defined before pooled analysis and was used both for the alcohol-risk contrasts and for the exercise and acute-stress positive-control contrasts.

Given the expected heterogeneity across region, perturbation structure, assay type, and time window, DerSimonian–Laird random-effects meta-analysis [57] was treated as the primary contrast-level summary for the alcohol-risk, exercise-positive-control, and stress-positive-control domains, whereas fixed-effect models were retained as complementary precision-weighted descriptions and for visualization. Heterogeneity was summarized using Cochran’s *Q*, a test of excess dispersion [58]; the associated *p* value; $I^{2}$, the percentage of observed variability attributed to between-contrast heterogeneity [59]; and $\tau^{2}$, the estimated between-contrast variance. Fixed-effect and random-effect estimates were compared with respect to pooled direction, confidence intervals, and significance classification. High-heterogeneity results were assigned to pre-specified reporting categories: concordant results with low or moderate heterogeneity were reported as model-consistent, non-significant summaries with substantial heterogeneity were described as directionally variable rather than simply null, and significant summaries with substantial heterogeneity were reported as heterogeneous associations rather than universal effects. Sign-consistency summaries were also calculated. Pre-specified subgroup summaries were generated across region family, assay family, contrast family, and quality tier. Leave-one-dataset-out analyses, rather than only leave-one-contrast-out analyses, tested whether any dataset materially altered the synthesis. Several contrast estimates nevertheless share a GEO series or parent study, and the conventional models do not fully account for that dependence. The pooled values are therefore descriptive summaries of aligned contrasts, not estimates of one universal alcohol effect or independent study replications.

### 4.8. Cell-Type Marker-Enrichment Analyses

To add a biological interpretation layer, canonical marker panels were defined for neuronal, excitatory, inhibitory, astrocytic, microglial, oligodendrocyte, oligodendrocyte precursor, and endothelial populations using established brain cell-type marker resources [60]. Within each dataset, expression values were standardized gene-wise, and marker-enrichment scores were calculated as the mean standardized expression across detected markers for each cell-type panel. These scores were interpreted as relative bulk marker-enrichment summaries rather than literal cellular deconvolution estimates.

Cell-type marker scores were compared across groups within each dataset using the same pairwise effect-size logic applied to the axis and composite-index framework. Cross-dataset synthesis of cell-type shifts was then performed using random-effects models as the primary pooled summary together with complementary fixed-effect estimates. Leave-one-out sensitivity analyses were additionally used to assess whether the strongest marker-level pooled shifts depended disproportionately on any single dataset. To relate the marker layer back to the primary transcriptomic framework, Spearman correlations were also computed between cell-type marker scores and the addiction, exercise, stress, ADI, ERI, and SAA metrics across datasets.

### 4.9. Dataset Quality Grading and Interpretation Boundaries

All screened datasets were assigned to evidence tiers using the same inclusion rubric applied during study assembly. Tier designations considered assay resolution, clarity of comparison structure, sample size, replication, and regional annotation. This grading scheme was used analytically to distinguish datasets that anchored the primary cross-study conclusions from those that primarily informed generalizability, positive-control behavior, or the limits of inference. The resulting inclusion, exclusion, and grading table is reported in Supplementary Table S10.

### 4.10. Software Environment

The computational workflow was implemented in the R statistical computing environment [61]. Data access and preprocessing used GEOquery [62], edgeR [41], and limma [63]; pathway scoring and pathway resources used GSVA [55], singscore [56], and msigdbr [64]; annotation and enrichment utilities used AnnotationDbi [43], org.Rn.eg.db [42], clusterProfiler [65], and ReactomePA [66]; and data handling and visualization used dplyr, tidyr, readr, ggplot2, and stringr through the tidyverse ecosystem [67]. Custom analysis scripts and reproducibility metadata supporting the analyses are available from the corresponding author upon reasonable request, consistent with the Data Availability Statement.

## 5. Conclusions

Pathway-level analysis can distinguish contrast-specific directional concordance from divergence across broader alcohol paradigms. In the closest matched pair, 24 h withdrawal-versus-control effects agreed for all three original axes and their three deterministic derived indices, whereas chronic-ethanol-versus-control effects agreed for only one of these six summaries. The derived indices are not independent replication tests. This distinction was stable to alternative scoring algorithms, pathway catalogs, and explicit reduction in pathway redundancy. The practical implication is that candidate AUD biomarkers or targets should be tied to a defined exposure or withdrawal state and should replicate across harmonized models, sexes, regions, species, and ultimately human tissue before being generalized.

## Figures and Tables

**Table 1:** Main-text summary of the focused matched-design analysis. Direction agreement counts the three original axes and three deterministic derived indices with the same effect direction in hippocampus and VTA. The Spearman rank correlation and median absolute difference compare the two six-summary Cohen’s *d* profiles. Because the indices are mathematical functions of the original axes, the six summaries are not interpreted as independent replication tests.

Contrast	Direction Agreement	Spearman ρ	Median |Δd|
24 h withdrawal vs. control	6/6	1.000	0.215
24 h withdrawal vs. chronic ethanol	4/6	0.486	0.867
Chronic ethanol vs. control	1/6	−0.371	1.165

**Table 2:** Experimental characteristics of the four primary rat datasets.

Accession	Region	Animals	Exposure and Tissue Collection	Groups Analyzed
GSE171051	Hippocampus	Adult male Sprague–Dawley rats (75–85 days)	Forced Lieber–DeCarli ethanol-containing liquid diet; chronic exposure followed by either immediate collection or 24 h withdrawal	Control diet, chronic ethanol, 24 h withdrawal; n=6 per group
GSE233921	VTA	Adult male Sprague–Dawley rats	Forced chronic ethanol liquid diet; immediate collection or 24 h withdrawal	Control diet, chronic ethanol, 24 h withdrawal; n=6 per group
GSE171400	Amygdala	Adult male Sprague–Dawley rats	Acute forced exposure, 1 g/kg ethanol intraperitoneally; collection after 1 h; two animals pooled per RNA sequencing (RNA-seq) sample	Saline control and acute ethanol; n=3 pooled RNA-seq samples per group
GSE73627	NAc	Adult male Wistar rats	Experiment 1: voluntary 6% ethanol/water access for 8 weeks. Experiment 2: 4 weeks of ethanol access, 3 weeks of daily intragastric water or ethanol gavage, then 2 additional weeks of access; immediate collection	Water (n=5), voluntary ethanol (n=5), water gavage (n=6), ethanol gavage (n=6)

## Data Availability

The public datasets analyzed or formally screened in this study are available from the Gene Expression Omnibus (GEO; https://www.ncbi.nlm.nih.gov/geo/ (accessed on 15 May 2026)) under accession numbers GSE171051, GSE233921, GSE171400, GSE73627, GSE112399, GSE49040, GSE49041, GSE60966, GSE31705, GSE299436, GSE126705, and GSE159136. GSE159136 was screened but excluded from the alcohol-domain analysis because a matched control group could not be recovered. Dataset roles, inclusion decisions, quality tiers, and processed results required to audit the analyses are consolidated in the Supplementary Tables file. No new raw sequencing data were generated in this study. Custom analysis scripts and reproducibility metadata supporting the analyses are available from the corresponding author upon reasonable request.

## Supplementary Materials

The following supporting information can be downloaded at https://www.mdpi.com/article/10.3390/ijms27198873/s1.

## Abbreviations

The following abbreviations are used in this manuscript:

ADI	Addiction-dominant index
ANOVA	Analysis of variance
AUD	Alcohol use disorder
BLA	Basolateral amygdala
BH	Benjamini–Hochberg
CeA	Central amygdala
CI	Confidence interval
DE	Differential expression
DEG	Differentially expressed gene
DNA	Deoxyribonucleic acid
ERI	Exercise-dominant index
FE	Fixed effect
FDR	False-discovery rate
GABA	Gamma-aminobutyric acid
GEO	Gene Expression Omnibus
GSE	GEO Series
GO:BP	Gene Ontology biological process
GSVA	Gene set variation analysis
$I^{2}$	Percentage of observed variability attributed to heterogeneity
MSigDB	Molecular Signatures Database
NAc	Nucleus accumbens
OPC	Oligodendrocyte precursor cell
PRISMA	Preferred Reporting Items for Systematic Reviews and Meta-Analyses
RE	Random effects
RNA-seq	RNA sequencing
SAA	Stress–activity balance
ssGSEA	Single-sample gene set enrichment analysis
TMM	Trimmed mean of M values
VTA	Ventral tegmental area
