# Supplementary material for: Pathway-Level Synthesis Reveals Contrast-Dependent Rat Brain Transcriptomic Responses to Alcohol
Source: Int J Mol Sci. 2026 Oct 5;27(19):8873. doi: 10.3390/ijms27198873 (PMC13665134; doi:10.3390/ijms27198873)

Supplementary Figure 1 | Alcohol−risk aligned effect sizes across datasets

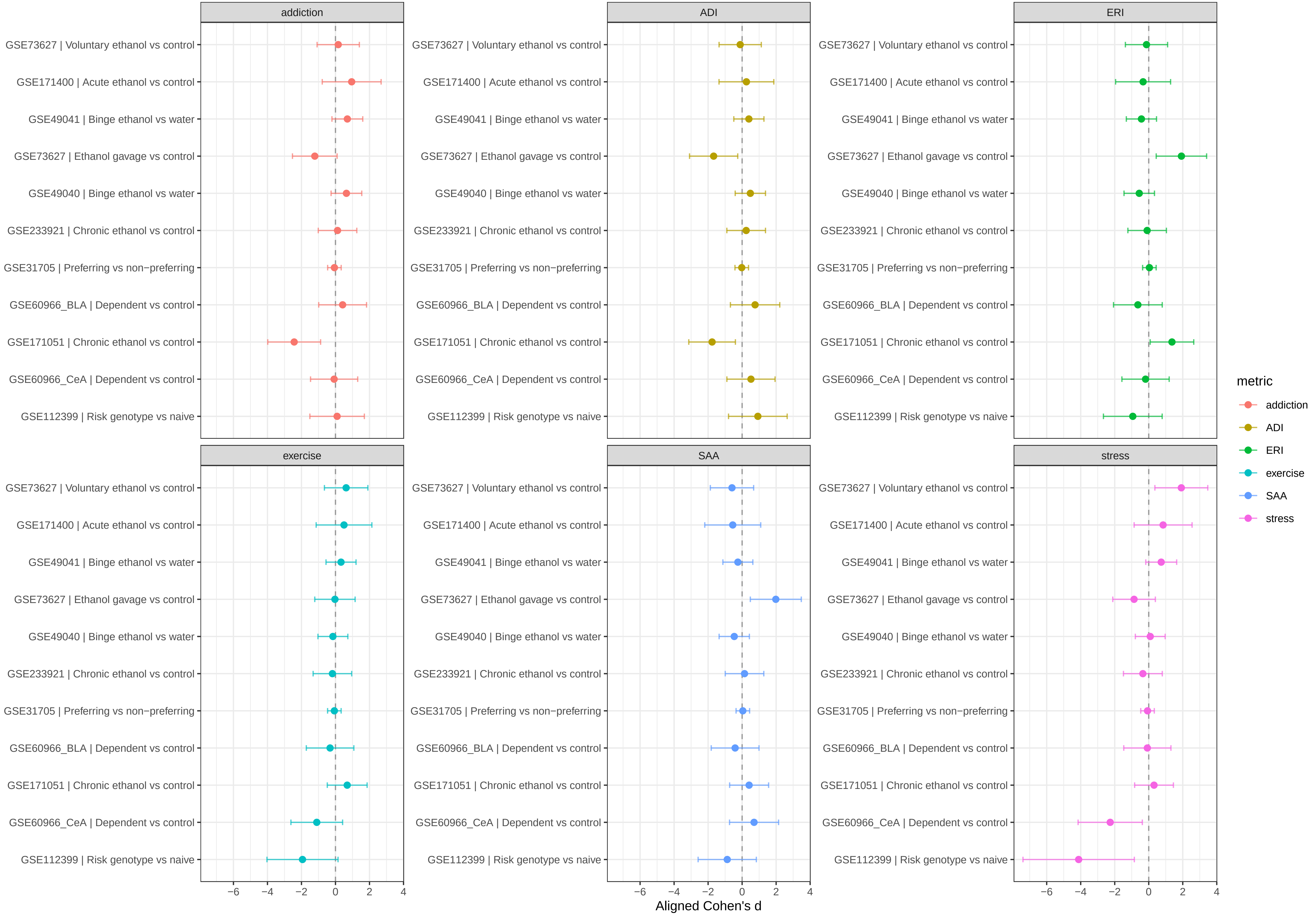

Supplement: Supplementary file 1 [file ijms-27-08873-s001.zip › supplementary figures/Supplementary_Figure_S1_Alcohol_Meta_Forest.pdf]
