# Supplementary material for: Pathway-Level Synthesis Reveals Contrast-Dependent Rat Brain Transcriptomic Responses to Alcohol
Source: Int J Mol Sci. 2026 Oct 5;27(19):8873. doi: 10.3390/ijms27198873 (PMC13665134; doi:10.3390/ijms27198873)
Supplement: Supplementary file 1 [file ijms-27-08873-s001.zip › supplementary figures/Supplementary_Figure_S7_Robustness_Sensitivities.pdf]

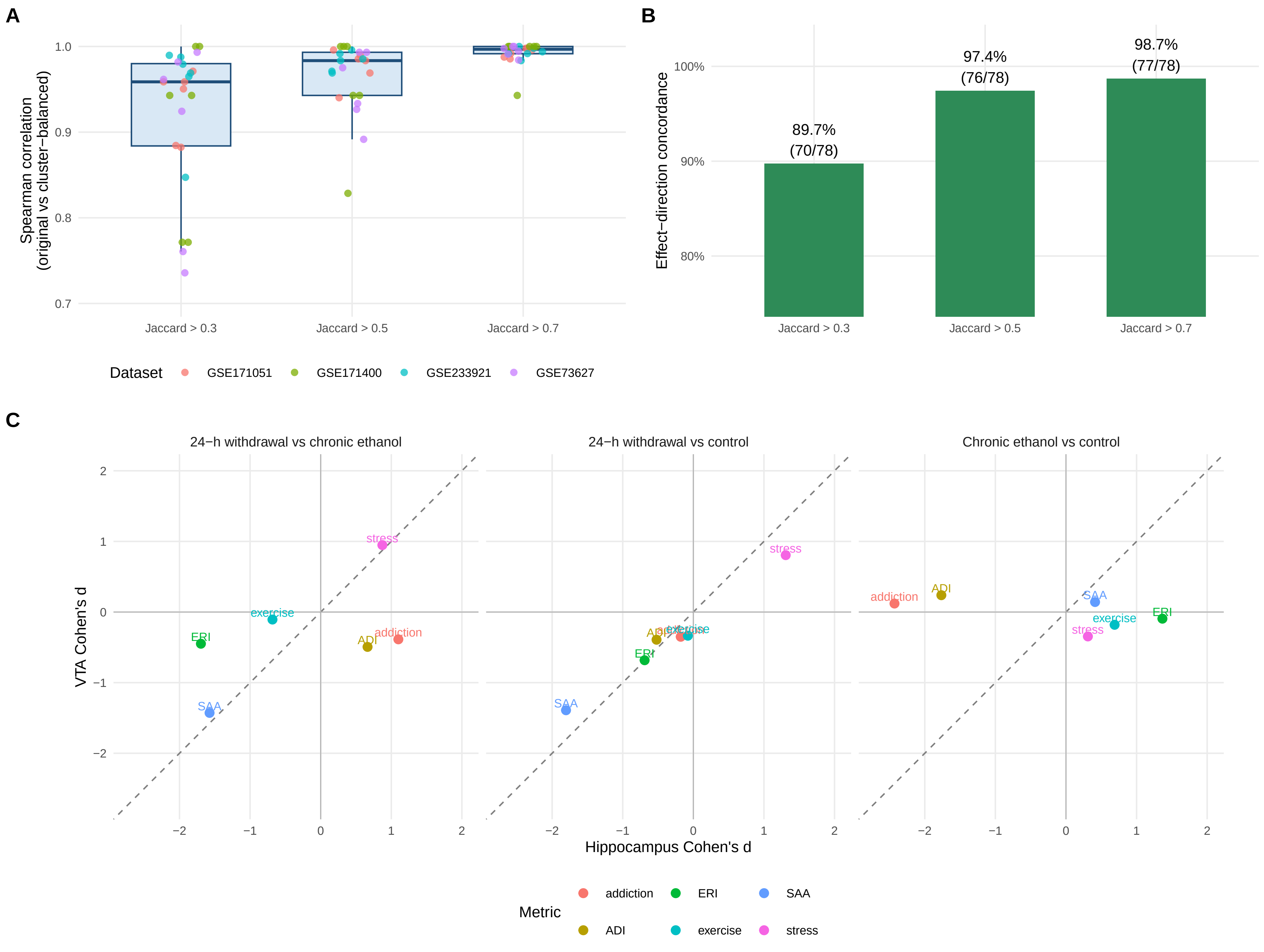

A
Spearman correlation
(original vs cluster−balanced)
1.0
0.9
0.8
0.7
Jaccard > 0.3
Jaccard > 0.5
Jaccard > 0.7
B
Effect−direction concordance
100%
90%
80%
89.7%
(70/78)
97.4%
(76/78)
98.7%
(77/78)
Jaccard > 0.3
Jaccard > 0.5
Jaccard > 0.7
Dataset
GSE171051
GSE171400
GSE233921
GSE73627
C
24−h withdrawal vs chronic ethanol
24−h withdrawal vs control
Chronic ethanol vs control
VTA Cohen's d
Hippocampus Cohen's d
stress
exercise
ERI
ADI
addiction
SAA
Metric
addiction
ERI
SAA
ADI
exercise
stress
