# Supplementary material for: Pathway-Level Synthesis Reveals Contrast-Dependent Rat Brain Transcriptomic Responses to Alcohol
Source: Int J Mol Sci. 2026 Oct 5;27(19):8873. doi: 10.3390/ijms27198873 (PMC13665134; doi:10.3390/ijms27198873)
Supplement: Supplementary file 1 [file ijms-27-08873-s001.zip › Supplementary Figure Legends.pdf]

Supplementary Figure Legends

Pathway-level synthesis reveals contrast-dependent rat brain transcriptomic responses to alcohol

Files supplied

The supplementary package contains one Excel data workbook and seven vector figures. All supporting tables, registries, search-audit information, and statistical outputs are consolidated in that workbook; no standalone table files or repeated supplementary Results narrative are supplied.

Supplementary Figure S1. Aligned alcohol-domain effect sizes

Contrast-level Cohen's d values for the three original pathway axes and three deterministic derived indices after orienting each contrast toward the alcohol-exposed, alcohol-dependent, alcohol-preferring, or alcohol-risk group. Error bars denote approximate 95% confidence intervals. The derived indices are mathematical functions of the original axes and are not independent outcomes.

Supplementary Figure S2. Domain-level pooled standardized effects

Precision-weighted fixed-effect pooled Cohen's d values for the addiction, exercise-positive-control, and stress-positive-control domains across the three original axes and three deterministic derived indices. Each cell reports the pooled effect estimate, the number of contributing contrasts (k), and I^2. These summaries describe domain-level directional patterns; random-effects estimates and confidence intervals are reported in Supplementary Table S6, and the derived indices are not independent outcomes.

Supplementary Figure S3. Scoring-method concordance

Median sample-level Spearman correlations among ssGSEA-, GSVA-, and singscore-derived axes and indices across the four primary datasets.

Supplementary Figure S4. Pathway-catalog sensitivity

Grouped mean index values after recalculating the primary datasets with Hallmark-only, Hallmark plus GO biological process, and broader combined pathway catalogs.

Supplementary Figure S5. Cell-marker synthesis

Fixed-effect and random-effects pooled marker-enrichment shifts for canonical neuronal, glial, vascular, and precursor panels. These are bulk marker summaries rather than cell-fraction estimates.

Supplementary Figure S6. Cell-marker and pathway-axis correlations

Median Spearman correlations between bulk cell-marker enrichment scores and the addiction, exercise, stress, ADI, ERI, and SAA metrics across datasets.

Supplementary Figure S7. Matched-design and pathway-redundancy sensitivity analyses

Panel A shows sample-level Spearman correlations between the original pathway axes and cluster-balanced axes after overlapping pathways were grouped at Jaccard thresholds of 0.3, 0.5, and 0.7. Panel B shows the proportion of pairwise effect directions preserved after cluster balancing. Panel

C compares the Cohen's d profiles for the three original axes and three deterministic derived indices between the closely matched hippocampal and VTA datasets for chronic ethanol versus control, 24-h withdrawal versus control, and 24-h withdrawal versus chronic ethanol. The derived indices are mathematical functions of the original axes and are not independent replication tests.
